# Thrombotic complications without evidence of hemolysis in paroxysmal nocturnal hemoglobinuria: is *eculizumab* indicated?

**DOI:** 10.1007/s00277-012-1511-5

**Published:** 2012-07-04

**Authors:** M. Bellido, V. H. J. van der Velden, F. W. G. Leebeek, P. A. W. te Boekhorst

**Affiliations:** 1 Department of Hematology, Erasmus Medical Center, Rotterdam, P.O. Box: 2040, 3000 DR Rotterdam, The Netherlands; 2 Department of Immunology, Erasmus Medical Center, Rotterdam, P.O. Box: 2040, 3000 DR Rotterdam, The Netherlands

**Fig. 1:**
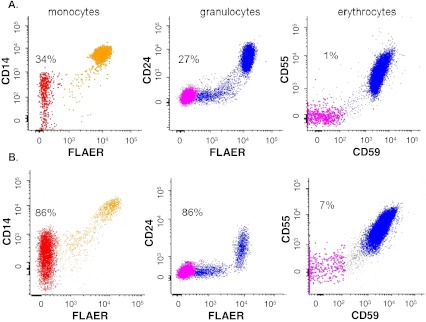
PNH clones at diagnosis (**a**) and their expansion (**b**) after treatment for SAA. Comparison of the PI-deficient clones at diagnosis of severe aplastic anemia (**a**) and after triple immunosuppression (**b**). Monocytes and granulocytes were gated based on scatter characteristics and expression of CD33 and CD45. PI deficiency was analyzed by FLAER and CD14 (monocytes, *left panel*) or FLAER and CD24 (granulocytes, *middle panel*). Erythrocytes (*right panel*) were gated based on scatter characteristics and PI deficiency was analyzed by CD59 and CD55. The *numbers* indicate the percentage of PI-deficient cells (type III completely lacking GPI-linked proteins) within the particular lineage. Minor populations of type II cells (partial lacking GPI-linked proteins) are present as well

Dear Editor,

We would like to discuss the treatment of paroxysmal nocturnal hemoglobinuria (PNH) by presenting the fatal outcome of one of our patients with this disease. A 30-year-old Caucasian man with a history of auto-immune hepatitis developed pancytopenia. He had no complaints and did not take any medication. On physical examination, a pale man was seen without any other remarkable findings. His hemoglobin was 8.3 g/dL (13.8–16.9 g/dL), leucocytes 0.30 (neutrophils 0.2) × 10^9^/L (3.5–10 × 10^9^/L), platelet count 16 × 10^9^/L (150–350 × 10^9^/L) and reticulocytes 8.4 × 10^9^/L (30–95 × 10^9^/L) without signs of hemolysis. Bone-marrow biopsy showed reduced cellularity (10 %) without any other (including cytogenetic) abnormalities. In peripheral blood, glycosylphosphatidylinositol (PI)-deficient clones were detected in 34, 27, and 1 % of the granulocyte, monocyte, and erythroid lineages, respectively. Severe aplastic anemia (SAA) was diagnosed secondary to PNH. Lacking an HLA-identical family donor, standard treatment with immunosuppression was started: rabbit anti-thymocyte globuline, methylprednisolone, and cyclosporine. This resulted in a partial response 3 months later: persistent transfusion-dependent anemia without signs of intravascular hemolysis, thrombocytopenia, and a normal leucocyte count. An expansion of the PI-deficient clones up to 86, 86, and 7 % of the granulocyte, monocyte, and erythroid lineages, respectively (Fig. 1) was noted.

Three months after initial presentation, he was re-admitted to hospital because of abdominal pain and bloody stools. Thrombosis of the superior mesenteric vein was diagnosed and anticoagulant therapy was instituted with nadroparin 15,200 IU/day subcutaneously. Eculizumab treatment was considered, but because of the complete absence of intravascular hemolysis, the effect of the anticoagulant therapy was awaited. Subsequently, he developed headache and a left hemiparesis. Repeated CT scans of the brain showed expanding ischemic areas parieto-occipital with secondary bleeding, and the patient finally died.

Thrombosis is the most feared and life-threatening complication in PNH patients, because after onset, it is frequently progressive and refractory to anticoagulant therapy [1, 2]. The mechanism of thrombotic complications in PNH is not completely understood, but it has mainly been attributed to intravascular hemolysis [1]. Experimental studies show that the free plasma hemoglobin liberated during hemolysis scavenges nitric oxide (NO). As a result, NO depletion produces dystonia and spasm of the smooth muscle, suppresses the anti-inflammatory effect on the endothelium and promotes platelet activation and thrombosis [3]. Eculizumab is a monoclonal antibody that binds complement factor C5, thereby inhibiting complement activation on erythrocytes and reducing hemolysis. As a result, eculizumab decreases thrombotic risks in PNH patients [2, 4, 5]. Interestingly, our patient suffered from progressive abdominal and cerebral thrombotic complications without any signs of intravascular hemolysis. It is also possible that the pathogenesis of thrombosis in PNH might be caused by other mechanisms associated to complement activation and not directly related to hemolysis [1, 6]. We speculate that the mechanism of thrombosis could be associated to platelet activation caused by the absence of CD59 and CD55 on the platelet surface [7, 8].

Internationally accepted indications for eculizumab treatment include transfusion-dependent hemolysis (four or more transfusions in 12 months) and PNH-related complications (i.e., thrombosis or renal failure) regardless of transfusion history [3]. Although no clinical data are available, eculizumab could be indicated in patients with thrombotic events in the absence of hemolysis [5, 9, 10].
